# Psychopathology and Gaming Disorder in Adolescents

**DOI:** 10.1001/jamanetworkopen.2025.28532

**Published:** 2025-07-29

**Authors:** Kylie Falcione, René Weber

**Affiliations:** 1 Department of Communication, Media Neuroscience Lab, University of California, Santa Barbara; 2 Department of Psychological and Brain Sciences, University of California, Santa Barbara; 3 Division of Communication and Media, Ewha Womans University, Seoul, Republic of Korea

## Abstract

**Importance:**

Although gaming disorder is recognized as a diagnosable behavioral addiction, uncertainty remains regarding its directional association with adolescent psychopathology. Clarifying this association is crucial for refining diagnostic frameworks and developing targeted interventions.

**Objective:**

To examine directional longitudinal associations between psychopathology and gaming disorder among adolescents using the Interaction of Person-Affect-Cognition-Execution model as a theoretical framework.

**Design, Setting, and Participants:**

This cohort study used data from the Adolescent Brain Cognitive Development Study (release 5.1), analyzing 4289 adolescents in the US who played video games and completed 3 waves of data collection (at ages 11-12, 12-13, and 13-14 years) between January 1, 2018, and December 31, 2022. Statistical analysis was performed from December 2024 to March 2025.

**Main Outcomes and Measures:**

Psychopathology was assessed using caregiver reports from the Child Behavior Checklist, which provided measures of depression, attention-deficit/hyperactivity disorder (ADHD), social problems, anxiety, and conduct disorder or aggression. Additional person-centered core characteristics (eg, negative life events, family conflict, bullying, and impulsivity) were incorporated. Gaming disorder was measured using the Video Game Addiction Questionnaire, which aligns with the *DSM-5* criteria for internet gaming disorder.

**Results:**

This cohort comprised 4289 adolescents (mean [SD] age, 168.8 [8.2] months; 2391 of 4288 [56%] males). Household income varied widely, with 1374 of 3877 households (35%) reporting an income from $100 000 to $199 000. Cross-lagged panel models (CLPMs) demonstrated that higher baseline levels of psychopathology were associated with an increased risk for subsequent gaming disorder from the 2-year to the 3-year follow-up (β = 0.03 [95% CI, 0.002-0.06]; *P* = .003) and from 3-year to the 4-year follow-up (β = 0.07 [95% CI, 0.04-0.10]; *P* < .001). Even when controlling for other personal core characteristics associated with increased risk, there was still a small to medium effect size of psychopathology associated with gaming disorder from the 3-year to the 4-year follow-up (β = 0.04 [95% CI, 0.002-0.07]; *P* = .04). In contrast, gaming disorder was not associated with later increases in psychopathology. Hierarchical mixed-effects models that accounted for both the panel structure and grouping of the data corroborated the results from the CLPMs.

**Conclusions and Relevance:**

The results of this cohort study suggest that psychopathology is significantly associated with the development of gaming disorder among adolescents. Clinical efforts to address underlying mental health issues, particularly for internalizing symptoms such as depression, anxiety, social problems, and ADHD, may reduce the incidence and severity of gaming disorder.

## Key Points

**Question:**

Is preexisting psychopathology associated with subsequent gaming disorder among adolescents, or is compulsive gaming associated with the development of psychopathology?

**Findings:**

In this cohort study of 4289 adolescents, longitudinal models revealed that higher baseline levels of psychopathology were significantly associated with an increased risk of developing gaming disorder 1 year later. However, there was no significant association between gaming disorder and the development or worsening of psychopathology.

**Meaning:**

These findings suggest that preexisting psychopathology is associated with the development of gaming disorder among adolescents.

## Teaser

This cohort study uses data from the Adolescent Brain Cognitive Development Study to examine directional longitudinal associations between psychopathology and gaming disorder among adolescents.

## Introduction

Video gaming is an integral aspect of adolescent life in the US, with recent estimates suggesting that up to 90% of adolescents engage in gaming for nearly 100 minutes per day.^1,2^ Digital games are designed with reward systems that sustain engagement,^3,4^ a feature that makes video games both highly enjoyable and addictive for adolescents,^5,6^ a developmental period characterized by heightened reward sensitivity and poor executive control.^7^ Despite the World Health Organization’s recognition of gaming disorder (GD) as a behavioral addiction in the *International Classification of Diseases, 11th Revision* (*ICD-11*),^8^ and its consideration in the American Psychiatric Association’s *Diagnostic and Statistical Manual of Mental Disorders* (Fifth Edition) (*DSM-5*),^9^ significant gaps remain in the understanding of the development of GD over time; 1 key variable of particular debate is that of psychopathology.

Gaming disorder is defined as impaired control over gaming behaviors, with increasing salience of gaming and significant negative consequences to daily functioning.^8,9^ Meta-analyses indicate that approximately 3% of global gamers meet these criteria, with prevalence rates increasing to 8.5% among adolescents.^10,11^ Both the *ICD-11* and *DSM-5* highlight the frequent co-occurrence of GD with other psychopathologies.^8,9^ Common comorbid psychopathologies include anxiety (92%), depression (89%), attention-deficit/hyperactivity disorder (ADHD; 85%), and social problems (75%),^12^ in addition to common externalizing psychopathologies such as conduct disorder^13,14^ and aggression.^15,16^

Although cross-sectional studies consistently reveal strong associations between GD and psychopathology,^17,18,19^ longitudinal investigations have produced mixed results.^20,21,22,23^ Some reviews suggest that GD exacerbates symptoms of depression, anxiety, and similar disorders, whereas others report that improvements in GD coincide with reductions in these symptoms. For instance, an early review from González-Bueso and colleagues^12^ posited that depression, anxiety, and social anxiety emerge as outcomes of problematic gaming. Similarly, Gentile and colleagues^20^ reported that worsening GD symptoms were associated with later increases in depression, anxiety, and social phobia; however, these findings did not account for preexisting psychopathology. More recent longitudinal studies have identified bidirectional associations between depressive symptoms and GD. Brunborg et al^13^ demonstrated a bidirectional relationship between depression and GD. These conflicting findings have led to divergent clinical recommendations, with some advocating for the direct treatment of gaming behaviors^13^ and others supporting early intervention for underlying mental health issues.^24,25,26,27^ Further complicating the issue, research focusing on ADHD and anxiety suggests that these conditions may have a particularly strong influence on the development of GD,^28,29,30,31,32^ implying that early treatment of ADHD and anxiety may help prevent the onset of problematic gaming. A review by Richard et al^33^ synthesized findings from 34 studies of GD across the lifespan, noting that psychopathologies such as depression, anxiety, or social problems, as well as conduct problems or aggression, seem to serve as an outcome of GD, whereas ADHD is primarily a cause of GD. Studies using multiple time points and larger samples, and accounting for baseline GD and psychopathology, such as those of Jeong et al,^34^ Teng et al,^35^ and Zhang et al,^36^ provide evidence that internalizing disorders, such as depression and anxiety, more reliably predict the emergence of GD rather than result from it.

The present study adopts a conceptual framework of the Interaction of Person-Affect-Cognition-Execution (I-PACE) model,^37,38^ one of the most widely used models to explain internet-related behavioral disorders. The I-PACE model posits that the development of GD depends on the interplay of biopsychological factors, personality traits, aversive social cognitions, and psychopathology. This model identifies psychopathology as a precursor of internet-related behavioral disorder; however, the I-PACE model also acknowledges the presence of other key personal core characteristics that also contribute to the development of behavioral disorders. To best understand whether psychopathology is a cause or consequence of GD, it is also necessary to account for the other contributing factors of GD, as outlined by the I-PACE model.

Biopsychological factors, such as genetic predispositions, negative life events, and sex, have been shown to foster maladaptive coping strategies and adversely affect learning and information processing.^39,40^ Moreover, male sex has emerged as a prominent correlate, with males being up to 4 times more likely than females to receive a diagnosis of GD.^41,42^ Personality traits, particularly impulsivity—a tendency to act on immediate urges without consideration of long-term consequences—are closely associated with deficits in executive control that underlie compulsive gaming behaviors.^43,44^ Individuals with deficits in inhibitory control are less able to manage emotional responses and delay gratification, leaning toward video games as a form of emotional coping, increasing their vulnerability to GD.^44,45^ Last, aversive social cognitions, such as family conflict and bullying, are also critical in understanding the development of GD, because family conflict and bullying may lead to media-based coping.^38,46,47,48^

**Figure 1.**
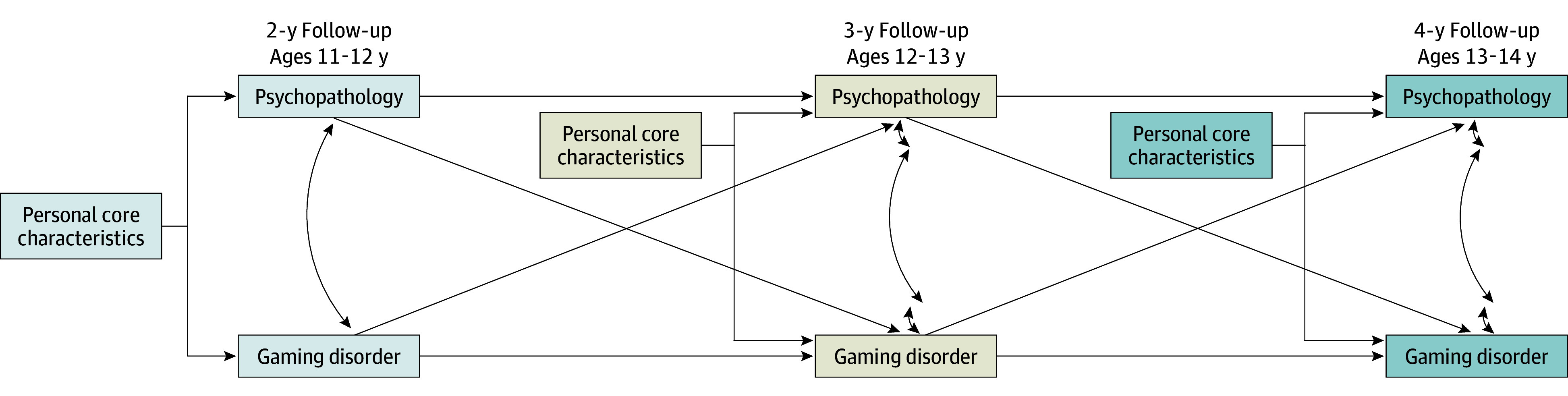
Conceptual Model Based on the I-PACE (Interaction of Person-Affect-Cognition-Execution) Model Person-Centered Risk Factors Psychopathology in the model is an unobserved variable with attention-deficit/hyperactivity disorder, anxiety, depression, social problems, and conduct disorder as observed indicators. Personal core characteristics include negative life events (biopsychological), impulsivity (personality), family conflict (social environment), and bullying (social environment).

Although the I-PACE model provides our theoretical framework, we take an exploratory approach to examine whether adolescents’ differences in psychopathology function primarily as a precursor to the development of GD or whether adolescents’ differences in GD are associated with differential psychopathologies over time, controlling for additional factors associated with GD (ie, biopsychological factors, personality traits, and aversive social cognitions). Thus, we suggest the conceptual cross-lagged panel model (CLPM) in Figure 1. Using structural equation models and hierarchical mixed-effects models (HMEMs) to control additional factors in the I-PACE model, we tested whether psychopathology functions primarily as a precursor to GD or whether it emerges as a consequence of problematic gaming behaviors. Ultimately, our findings are expected to inform clinical practice by identifying the most effective targets for early intervention, thereby optimizing treatment outcomes for adolescents at risk for GD.

## Methods

### ABCD Data

This cohort study uses data from January 1, 2018, to December 31, 2022, from the Adolescent Behavioral Cognitive Development (ABCD) Study dataset release 5.1 (released in July 2023),^49^ which we downloaded using NDA Tools.^50^ This study followed the Strengthening the Reporting of Observational Studies in Epidemiology (STROBE) reporting guideline. The ABCD Study is a nationally representative adolescent panel study of 11 572 youths examining teen development through questionnaires, behavioral tasks, and neurologic markers. Written informed consent was obtained from parents and assent from children by ABCD investigators at each time point. A centralized review board approved all procedures at the University of California, San Diego.^51^ Our institution did not require additional institutional review board approval for this study. We did, however, fully comply with all National Institutes of Health requirements for accessing and using the data, which included obtaining institutional approval via the National Institutes of Health Request Access process.

### Participants

Participants in the ABCD Study underwent a clinical and behavioral assessment every year. The GD questionnaires were introduced in the ABCD dataset starting from the 2-year follow-up.^40,51^ Thus, the present study included adolescents who completed assessments at the 2-year (ages 11-12 years; 2018-2020), 3-year (ages 12-13 years; 2019-2021), and 4-year (ages 13-14 years; 2020-2022) ABCD Study follow-ups. Due to the partial completion of the 4-year data wave, the initial sample was reduced to 4754 participants. From there, we further excluded participants who never reported any gaming across the 3 time points, resulting in our final sample of 4289 adolescents. Race and ethnicity categories, reported by parents and determined by the ABCD Consortium, included Asian, Black, Hispanic, White, and other (Native American, Alaska Native, Native Hawaiian, Guamanian, Samoan, other race, multiple races, or refused to answer).^52^ The sample reflects the demographic characteristics in Table 1.

**Table 1.** Participants’ Demographic Information

Characteristic	Participants, No./total No. (%)			
	2-y Follow-up (n = 10 973)	3-y Follow-up (n = 10 336)	4-y Follow-up (n = 4754)	Final sample (n = 4289)^a^
Age, mean (SD), mo	144.3 (8.0)	155.0 (7.8)	169.0 (8.2)	168.8 (8.2)
Missing	1	1	0	0
Household income, $				
<34 999	1756/10 009 (18)	1480/9267 (16)	593/4280 (14)	545/3877 (14)
35 000-49 999	731/10 009 (7)	649/9267 (7)	301/4280 (7)	271/3877 (7)
50 000-74 999	1314/10 009 (13)	1135/9267 (12)	500/4280 (12)	466/3877 (12)
75 000-99 999	1374/10 009 (14)	1299/9267 (14)	589/4280 (14)	537/3877 (14)
100 000-199 999	3391/10 009 (34)	3247/9267 (35)	1519/4280 (35)	1374/3877 (35)
≥200 000	1443/10 009 (14)	1457/9267 (16)	778/4280 (18)	684/3877 (18)
Missing	964	1069	474	412
Parental educational level				
<High school	630/10 888 (6)	550/10 079 (6)	245/4671 (5)	230/4223 (5)
High school graduate	837/10 888 (8)	733/10 079 (7)	310/4671 (7)	285/4223 (7)
GED certificate or equivalent	288/10 888 (3)	229/10 079 (2)	94/4671 (2)	83/4223 (2)
<1 y of College	418/10 888 (4)	404/10 079 (4)	188/4671 (4)	170/4223 (4)
≥1 y of College, no degree	1273/10 888 (12)	1072/10 079 (11)	486/4671 (10)	450/4223 (11)
Associate degree: technical	789/10 888 (7)	713/10 079 (7)	328/4671 (7)	306/4223 (7)
Associate degree: academic	629/10 888 (6)	586/10 079 (6)	273/4671 (6)	252/4223 (6)
Bachelor’s degree	3090/10 888 (28)	2933/10 079 (29)	1410/4671 (30)	1253/4223 (30)
Master’s degree	2202/10888 (20)	2172/10 079 (22)	1006/4671 (22)	898/4223 (21)
Professional school degree: MD, DVN, JD	329/10 888 (3)	311/10 079 (3)	147/4671 (3)	133/4223 (3)
Doctoral degree	403/10 888 (4)	376/10 079 (4)	184/4671 (4)	163/4223 (4)
Missing	85	257	83	66
Sex				
Female	5216/10 969 (48)	4908/10 332 (48)	2265/4753 (48)	1897/4288 (44)
Male	5753/10 969 (52)	5424/10 332 (52)	2488/4753 (52)	2391/4288 (56)
Missing	4	4	1	1
Race and ethnicity				
Asian	231/10 971 (2)	221/10 334 (2)	110/4754 (2)	96/4289 (2)
Black	1561/10 971 (14)	1361/10 334 (13)	509/4754 (11)	471/4289 (11)
Hispanic	2164/10 971 (20)	2075/10 334 (20)	979/4754 (21)	889/4289 (21)
White	5860/10 971 (53)	5591/10 334 (54)	2682/4754 (56)	2408/4289 (56)
Other^b^	1155/10 971 (11)	1086/10 334 (11)	474/4754 (10)	425/4289 (10)
Missing	2	2	0	0

Abbreviation: GED, General Educational Development.

^a^ The final sample accounts for all of the individuals who were present during all 3 waves of data collection and met our criteria of playing video games for at least 1 of the 3 time points.

^b^ The other race category includes Native American, Alaska Native, Native Hawaiian, Guamanian, Samoan, other race, multiple races, or refused to answer.

### Measures and Data Preparation

#### GD and Psychopathology

Gaming disorder was measured using the Video Game Addiction Questionnaire,^40^ which aligns with *ICD-11* and *DSM-5*^53^ criteria and assesses 6 dimensions: preoccupation, tolerance, escape, loss of control, withdrawal, and risk opportunities (score range, 6-36, where higher scores indicate greater symptoms of GD). Psychopathology was operationalized as a latent trait from 5 Child Behavior Checklist subscales^54,55,56,57^ completed by caregivers: depression (score range, 0-24), ADHD (score range, 0-14), social problems (score range, 0-18), anxiety (score range, 0-18), and conduct disorder (score range, 0-21), with higher scores indicating higher symptom severity for all subscales.

#### Personal Core Characteristics

The biopsychological constitution facet of the I-PACE model was conceptualized as negative life events to best represent early life stressors and sex. Negative life events were operationalized using the Adverse Life Events Scale, which assesses stress exposure based on youth self-report.^54^ Teens were asked to report on life events within the past year and report if they were mostly good or mostly bad. Examples include “Family member had a drug/alcohol problem?” or “Someone in the family died?”^54^ A sum score (range, 0-51) was then created based on how many mostly bad events were reported, with higher scores indicating more negative life events. Sex was collected through parent self-report.

Social cognitions from the I-PACE model included family conflict and bullying. Family conflict was assessed using the Family Environment Scale, specifically the family conflict subscale of the measure (eg, “We fight a lot in our family”), with a score range of 0 to 9, where higher scores indicated more family conflict.^58^ Bullying (eg, “Some kids left me out of an activity I wanted to join”) was measured through the youth-reported Peer Experiences Questionnaire (score range, 9-45, where higher scores indicated more bullying).^59^

Impulsivity was selected as a key personality trait associated with the development of GD. Impulsivity was assessed using the Urgency–Premeditation-Perseverance–Sensation Seeking–Positive Urgency (UPPS-P) scale, measuring 5 dimensions of impulsivity (ie, negative and positive urgency, sensation seeking, and lack of perseverance and premeditation). A shortened version of the UPPS-P scale was used in the ABCD Study to be more youth appropriate^54^ (eg, “When I feel rejected, I often say things that I later regret”). Higher total youth-reported scores (range, 20-77) reflect elevated levels of impulsivity. Impulsivity was assessed only at the 2-year and 4-year follow-up visits.

### Statistical Analysis

Statistical analysis was performed from December 2024 to March 2025. To examine the directional associations between psychopathology and GD across 3 time points, each variable was examined for normality, and if it did not meet that assumption, it was log transformed. We fit several longitudinal structural equation models. We first examined our factor loadings for psychopathology using confirmatory factor analysis, followed by estimating a traditional CLPM to test the temporal associations between a latent psychopathology factor and GD symptoms, controlling for sex. Last, we ran the CLPM, adding in the personal core characteristic variables in a subsequent analysis. Following recommendations by Hamaker et al^60,61^ and Orth et al,^62^ we then compared this approach with a random-intercept CLPM. Conceptually, the CLPM is a better-suited model as our primary research question focuses on between-person associations over time (ie, whether differences in psychopathology are associated with adolescents’ rank order in GD longitudinally).^60^ In addition, we controlled for stable traitlike variables (impulsivity, negative life events, family conflict, bullying, and sex) to reduce potential confounding associations of unmeasured traits in the autoregressive paths.

Model comparison between CLPM (model fit: comparative fit index [CFI] = 0.95; root mean square error of approximation [RMSEA] = 0.04; standardized root mean square residual [SRMR] = 0.05) and random-intercept CLPM (model fit: CFI = 0.92; RMSEA = 0.06; SRMR = 0.06) revealed no significant difference (χ^2^ test difference, 1012.68; *P* ≥ .99), although descriptively the CLPM demonstrated a slightly better fit across multiple indices, supporting our conceptually driven model selection. All models were estimated using maximum likelihood using standardized β coefficients with robust standard errors in the lavaan^63^ package in R, version 4.4.2 (R Project for Statistical Computing). Full-information maximum likelihood was used to handle missing data on the outcome measures. Model fit was evaluated using the Hu and Bentler^64^ markers of good model fit (CFI ≥0.95; RMSEA ≤0.06; and SRMR ≤0.08). We tested measurement invariance through nested model comparisons examining configural, metric, and scalar invariance and also estimated individual CLPMs for each psychopathology indicator.

As a robustness check, we estimated HMEMs, treating the 21 research sites as a random effect to account for potential clustering. These models allowed us to examine our research questions within a multilevel framework as others have done using ABCD Study data (eg, He and colleagues^65^), while controlling the nested structure of the data.

In accordance with benchmarks from a recent meta-analysis by Orth and colleagues^66^ examining the effect sizes for 1028 CLPMs across various fields of study, we identified β = 0.03 (25th percentile) as a small effect size, β = 0.07 (50th percentile) as a medium effect size, and β = 0.12 (75th percentile) as a large effect size for CLPMs. A 2-sided *P* < .05 was considered statistically significant.

## Results

### Data Characteristics and Correlations

The sample of 4289 participants, representative of the ABCD Study sample, comprised 2391 of 4288 male adolescents (56%) and 1897 of 4288 female adolescents (44%) and 96 Asian adolescents (2%), 471 Black adolescents (11%), 889 Hispanic adolescents (21%), and 2408 White adolescents (56%) (Table 1). Household income varied widely, with 1374 of 3877 households (35%) reporting an income from $100 000 to $199 000. Most variables were right skewed, as expected in a nonclinical sample, and were log transformed. Only GD, ADHD symptoms, family conflict, and impulsivity met normality assumptions. Given the nonnormal distribution, rank-based Spearman correlations were used. Psychopathology measures were strongly correlated (from *r* = 0.30 to *r* = 0.79). Descriptive statistics (Table 2)^53^ included gaming time and GD risk.^53^ Supplemental materials and analyses can be found on Open Science Framework.^67^

**Table 2.** Descriptive Statistics

Characteristic^a^	Mean (SD)		
	2-y Follow-up (n = 4289)	3-y Follow-up (n = 4289)	4-y Follow-up (n = 4289)
Gaming disorder score	10.4 (5.9)	12.2 (6.4)	11.7 (6.2)
Minutes gaming per week	127.4 (170.8)	156.6 (192.9)	186.1 (215.5)
Social problems	1.3 (2.0)	1.2 (2.0)	1.0 (1.8)
Depression	1.5 (2.2)	1.7 (2.5)	1.8 (2.7)
ADHD	2.3 (2.8)	2.3 (2.8)	2.1 (2.6)
Conduct disorder	1.1 (2.1)	1.1 (2.1)	1.1 (2.1)
Anxiety	1.8 (2.3)	1.8 (2.4)	1.7 (2.3)
Family conflict	1.9 (1.8)	2.1 (2.0)	2.2 (2.1)
Negative life events	4.8 (5.2)	4.3 (4.7)	4.0 (4.6)
Impulsivity	39.3 (7.8)	41.2 (5.1)	41.5 (7.8)
Gaming disorder risk, No./total No. (%)^b^			
Low	3736/4289 (87)	2982/4289 (70)	3086/4289 (72)
High	553/4289 (13)	1307/4289 (30)	1203/4289 (28)

Abbreviation: ADHD, attention-deficit/hyperactivity disorder.

^a^ Gaming disorder score: range, 6 to 36, with higher scores indicating greater gaming disorder symptoms; social problems: range, 0 to 18, with higher scores indicating greater social problems; depression: range, 0 to 24, with higher scores indicating greater depression symptoms; ADHD: range, 0 to 14, with higher scores indicating greater ADHD symptoms; conduct disorder: range, 0 to 21, with higher scores indicating more conduct disorder symptoms; anxiety: range, 0 to 18, with higher scores indicating more anxiety symptoms; family conflict: range, 0 to 9, with higher scores indicating greater family conflict; negative life events: range, 0 to 51, with higher scores indicating more negative life events; impulsivity: range, 20 to 77, with higher scores indicating greater impulsivity.

^b^ Gaming disorder risk is based on a suggested cutoff score set to more than 3 on at least 4 of the 6 criteria (polythetic scoring).^53^

### Overall CLPM

**Figure 2.**
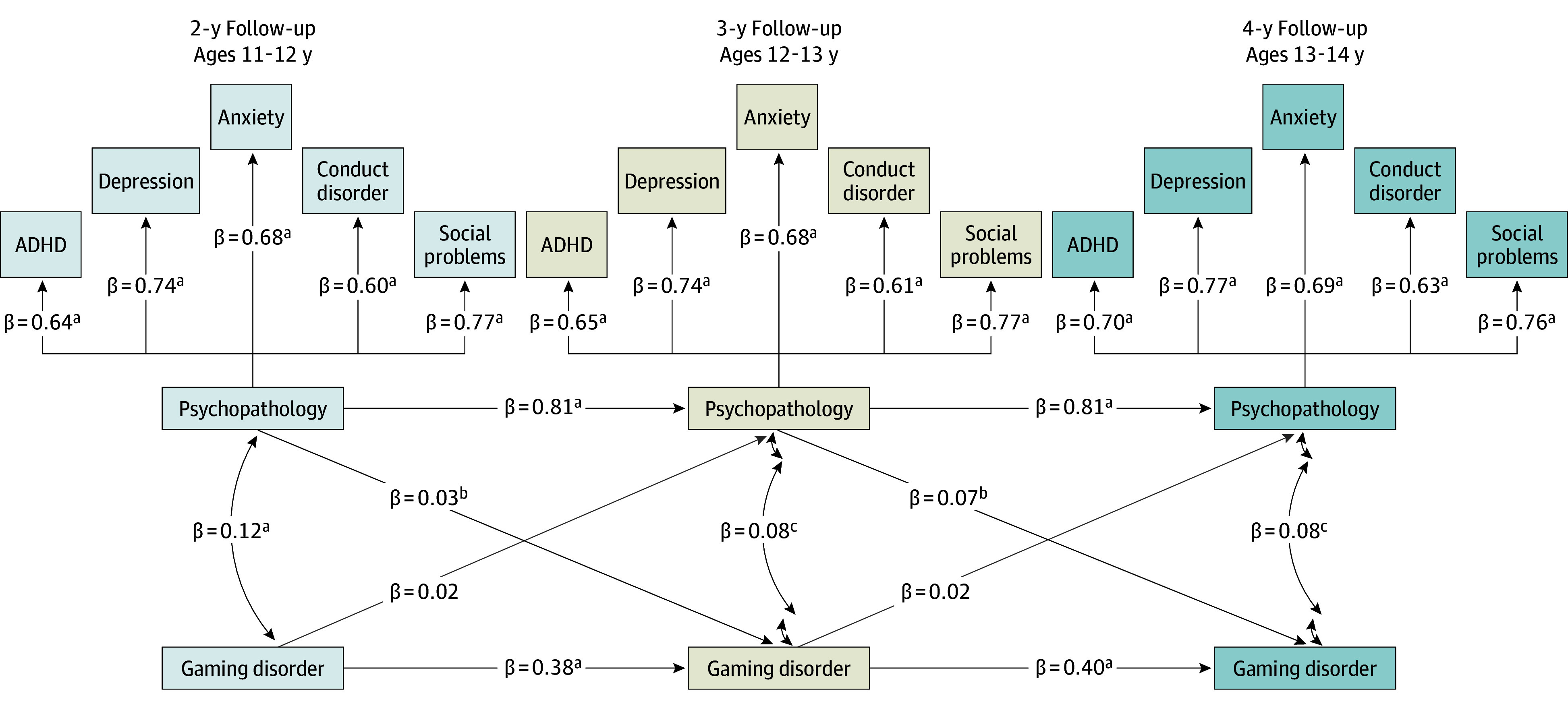
Observed Model Without Controlling for Personal Core Characteristics The model’s fit statistics are comparative fit index = 0.96; root mean square error of approximation = 0.06; and standardized root mean square residual = 0.05. All results are reported using standardized β coefficients. ADHD indicates attention-deficit/hyperactivity disorder. ^a^*P* < .001. ^b^*P* < .05. ^c^*P* < .01.

**Figure 3.**
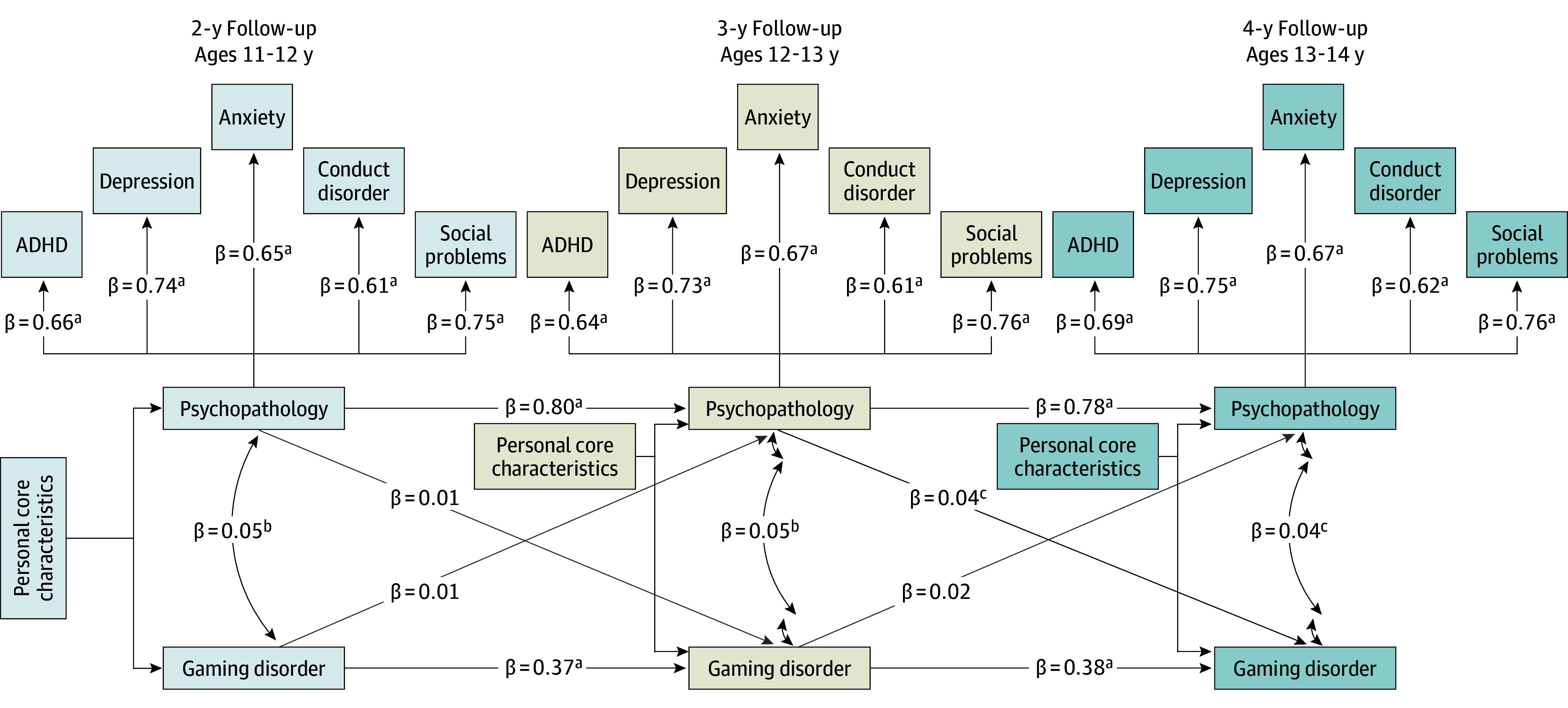
Observed Model With Controlling for Personal Core Characteristics The model’s fit statistics are comparative fit index = 0.95; root mean square error of approximation = 0.04; and standardized root mean square residual = 0.05. All results are reported using standardized β coefficients. ADHD indicates attention-deficit/hyperactivity disorder. ^a^*P* < .001. ^b^*P* < .05. ^c^*P* < .01.

The confirmatory factor analysis confirmed that depression, anxiety, conduct disorder, social problems, and ADHD symptoms were highly and significantly correlated with 1 main psychopathology factor across all 3 time points (2 years: ADHD, β = 0.66 [95% CI, 0.63-0.68]; conduct disorder, β = 0.62 [95% CI, 0.59-0.65]; depression, β = 0.76 [95% CI, 0.74-0.78]; anxiety, β = 0.69 [95% CI, 0.67-0.71]; and social problems, β = 0.80 [95% CI, 0.78-0.82]; 3 years: ADHD, β = 0.66 [95% CI, 0.64-0.68]; conduct disorder, β = 0.62 [95% CI, 0.59-0.64]; depression, β = 0.76 [95% CI, 0.74-0.78]; anxiety, β = 0.71 [95% CI, 0.68-0.73]; and social problems, β = 0.80 [95% CI, 0.77-0.82]; 4 years: ADHD, β = 0.67 [95% CI, 0.65-0.70]; conduct disorder, β = 0.63 [95% CI, 0.60-0.66]; depression, β = 0.77 [95% CI, 0.75-0.79]; anxiety, β = 0.71 [95% CI, 0.69-0.73]; and social problem, β = 0.79 [95% CI, 0.78-0.81]), with social problems and depression consistently showing the strongest loadings (Figure 2 and Figure 3).

Next, we examined the directional associations between psychopathology and GD across the 3 time points, controlling for sex. From the 2-year to 3-year visit, psychopathology was significantly associated with an increase in GD (β = 0.03 [95% CI, 0.002-0.06]; *P* = .003); similarly, in the 3-year follow-up, psychopathology was significantly associated with GD (β = 0.07 [95% CI, 0.04-0.10]; *P* < .001) at the 4-year follow-up. In the other direction, GD was not significantly associated with psychopathology at either time point (Figure 2).

Next, personal core characteristics were included as potential confounders and regressed on both the psychopathology factor and GD, again controlling for sex. The initial CLPM demonstrated moderate to poor fit (CFI = 0.71; RMSEA = 0.10; SRMR = 0.06). Given the conceptual expectation that specific psychopathology indicators would show stability over time beyond what is captured by the latent factors, we implemented equality constraints on the error variances of observed psychopathology indicators across time points within each latent factor. After implementing these theoretically motivated constraints, the revised model achieved good fit (CFI = 0.95; RMSEA = 0.04; SRMR = 0.05). Psychopathology in the 3-year follow-up was significantly associated with GD 1 year later (β = 0.04 [95% CI, 0.002-0.07]; *P* = .04); however, psychopathology from the 2-year to the 3-year follow-up was not significantly associated with GD (β = 0.01 [95% CI, –0.03 to 0.04]; *P* = .71). Conversely, GD remained an insignificant factor associated with psychopathology. Figure 3 presents the observed model with the hypothesized I-PACE model risk factors.

In addition to the CLPM, we estimated 2 HMEMs that accounted for both the panel structure and the grouping of the data, mirroring a recent similar analysis using the ABCD Study dataset,^65^ in which participants were grouped into families and into research sites. The best-fit model across multiple fit indices indicated that psychopathology is associated with GD over time and explained 21.2% of the GD variance. The pattern of the observed associations across the 3 time points suggests that psychopathology is a significantly associated antecedent of GD and not a significantly associated outcome.

### CLPMs of Individual Psychopathologies

We also analyzed each of the 5 psychopathology indicators separately to understand which of them may be the primary factors associated with GD. Depression (β = 0.05 [95% CI, 0.03-0.08]; *P* < .001), social problems (β = 0.04 [95% CI, 0.006-0.06]; *P* = .02), and anxiety (β = 0.05 [95% CI, 0.02-0.08]; *P* = .049) at 3-year follow-up were associated with GD at 4-year follow-up. ADHD symptoms were associated with GD from the 2-year to 3-year follow-up (β = 0.06 [95% CI, 0.3-0.08]; *P* = .02), but not the following year (β = 0.03 [95% CI, –0.002 to 0.06]; *P* = .06). Conduct disorder showed no significant associations with GD. For all models, GD was not significantly associated with psychopathology 1 year later.

## Discussion

In this cohort study of adolescents, our findings indicated that preexisting psychopathology was significantly associated with the subsequent development of GD, while GD did not appear to be associated with later increases in psychopathologies, even after accounting for personal core characteristics and controlling for sex. Internalizing disorders, in particular, were associated with the development of subsequent GD, with depression symptoms being the strongest factor, followed by anxiety, ADHD, and social problems.^18,68^ In contrast to studies that have suggested a bidirectional relationship,^18,19,20,21,22^ our CLPM and HMEMs, designed to account for baseline levels of psychopathology and the hierarchical, dependent structure of the data, did not reveal reciprocal associations. However, our analyses were limited to only 3 time points. Future research should examine within-person effects to more accurately identify potential bidirectional or “downward spiral” associations, which the present study cannot fully rule out.^68,69,70,71^ Our CLPMss were all able to reach good model fit with the theoretically motivated constraints.^64^

At first sight, the effect sizes in our models seem small and, as such, of minor practical relevance. However, according to recently developed effect size guidelines for CLPMs,^66^ the standardized coefficients of β = 0.07 and β = 0.04 for the main cross-lagged associations correspond with effect sizes at the 51st and 31st percentiles, respectively, of other CLPMs in related fields.

The present study refines our understanding of the association between psychopathology and compulsive gaming behavior within the theoretical framework of the I-PACE model.^37,38^ Our findings support the model’s assertion that psychopathological processes are critical antecedents to behavioral addictions.

### Clinical Implications

Our findings suggest that early identification and treatment of mental health conditions, particularly depression and anxiety, may serve as primary prevention strategies for GD in adolescents. Clinically, this finding translates to incorporating screenings for problematic media use during mental health assessments of adolescents with internalizing symptoms, using validated tools such as the 7-item Gaming Addiction Scale^72^ or the Problematic Media Use Measure.^73^ Treatment should prioritize addressing underlying mental health conditions as the primary therapeutic target, with cognitive-behavioral therapy protocols for adolescent depression and anxiety modified to include gaming-specific modules when necessary.

### Limitations

Despite using a large, nationally semirepresentative sample and a longitudinal perspective in our analyses, certain limitations warrant mention. For instance, measures of psychopathology in the ABCD Study dataset were parental self-reports on their child. A study by Verhulst and van der Ende^74^ indicated that parents underestimate their adolescents’ psychopathology symptoms when compared with their child’s self-reports, suggesting that psychopathology symptoms for this study may be underreported. Furthermore, the measures of psychopathology were not based on formal clinical interviews.^75^ Similarly, GD was measured with 6 Likert-type scales covering core tenets of GD based on the 9 *DSM-5* criteria for internet GD. However, more clinically relevant measures of GD^76^ that have been validated in clinical populations^72^ are available.

Video games rank among adolescents’ most preferred activities, covering 56% of their screen time,^77^ with 40% of adolescents playing video games daily.^78^ The ABCD Study lacks detailed gaming measurements, relying on screen time rather than game-specific factors such as content, motivation, or social context (eg, single-player vs multiplayer games).^79,80^ This limitation may underestimate the true associations of gaming behaviors with adolescent psychopathology, as screen time alone cannot capture the complexity of gaming experiences.

Despite the shortcomings in the ABCD Study data and the resulting increased error in measures, we still observed small to medium effect sizes^66^ in our models. Although we can only speculate at this time, we expect even larger associations of psychopathology with excessive, compulsive video game playing when refined measures of video game content and adolescents’ media use behaviors are used in future studies.

## Conclusions

In this cohort study of more than 4000 adolescents, our findings suggest that preexisting psychopathology was significantly associated with the subsequent development of GD, while GD was not associated with later increases in psychopathologies, even after accounting for personal core characteristics and controlling for sex. Overall, our findings contribute to a growing body of literature that emphasizes the primacy of preexisting mental health conditions in the development of GD, thereby reinforcing the importance of early, targeted interventions by clinicians. Future research should incorporate additional follow-up assessments, use clinical diagnostic interviews to validate self-report measures, and use state-of-the-art measures of media content and adolescents’ media use motivations and behaviors.

## Notes

Supplement.
Data Sharing Statement
