# Supplementary material for: Psychopathology and Gaming Disorder in Adolescents
Source: JAMA Netw Open. 2025 Jul 29;8(7):e2528532. doi: 10.1001/jamanetworkopen.2025.28532 (PMC12308444; doi:10.1001/jamanetworkopen.2025.28532)
Supplement: Supplement. — Data Sharing Statement [file jamanetwopen-e2528532-s001.pdf]

# Data Sharing Statement

Falcione. Psychopathology and Gaming Disorder in Adolescents. *JAMA Netw Open*. Published July 29, 2025. doi:10.1001/jamanetworkopen.2025.28532

**Data**
**Data available:** No

**Additional Information**
**Explanation for why data not available:** The data used is part of the NIMH Adolescent Brain Cognitive Development (ABCD) Data Use Agreement. As such, we are not allowed to share the data. However, investigators can independently request the data from the NIMH. We will make all analytical protocols, code, and everything else needed to replicate our analyses available.
